# Emergency Department Utilization and Patient Acuity in the Setting of Care-Seeking Hesitancy: Insights from the COVID-19 Pandemic

**DOI:** 10.5811/westjem.43530

**Published:** 2025-09-25

**Authors:** Eric Frazier, Nouri Modallalkar, Natassia Dunn, Bharath Chakravarthy, Luis Gonzales, Soheil Saadat

**Affiliations:** * University of California, Irvine, School of Medicine, California; † Independent Researcher, San Jose, California; ‡ Mount Sinai West, Department of Obstetrics and Gynecology, New York, New York; § University of California, Irvine, Department of Emergency Medicine, California; || University of California Irvine Medical Center, Orange, California

## Abstract

**Introduction:**

The coronavirus disease 2019 (COVID-19) pandemic significantly altered emergency department (ED) utilization patterns. This study quantifies the statistics at a Level I trauma center in Southern California from 14 months before to nine months after the start of the pandemic (January 2019–December 2020). We hypothesized that during the COVID-19 pandemic, changes in ED use patterns impacted patient acuity, as measured by admission rate, mortality rate, ED volume, Emergency Severity Index (ESI), and female:male ratio, even when controlling for COVID-19 cases.

**Methods:**

In this study we examined 97,793 ED visits from January 2019–December 2020 at the University of California, Irvine Medical Center in Orange, CA, via an administrative database comprised of anonymized datapoints from the electronic health record. We included all months from January 2019–December 2020 to account for potential secular trends by calendar month. Primary outcome measures were hospital admission rate and all-causes mortality rate among non-COVID-19 patients who presented to the ED. Secondary outcome measures included the mean number of ED visits per month, mean ESI, and female:male ratio among non-COVID-19 patients. Statistical analyses were performed.

**Results:**

We found an increase in the mortality rate per ED visit of 0.8859% before the pandemic to 1.2706% (P < .001) during the pandemic. After excluding COVID-19 cases, the mortality rate per ED visit remained elevated at 1.1746% (P < .001), a relative increase of 32.6%. Hospital admission rate increased from 26.0% before the pandemic to 32.3% during the pandemic (P < .001). The mean number of ED visits per month decreased from 4,271.2 ± 193.1 before the pandemic to 3,558.7 ± 437.1 per month during the pandemic (P < .001), a relative decrease of 16.7% when excluding COVID-19 cases. The mean ESI of non-COVID-19 related cases during the pandemic decreased from 2.85 pre-pandemic to 2.84 during the pandemic (P = .03). The female:male ratio decreased from 1.003 pre-pandemic to 0.885 during the pandemic (P < .001).

**Conclusion:**

This study reveals a decrease in patient volume with an increase in mortality and admission rate, demonstrating an association between shifts in ED utilization patterns and increased patient acuity during the pandemic. Understanding patients’ emergency care-seeking behavior during this period is essential for preparing for future large-scale public health crises and optimizing ED resource allocation and mobilization based on lessons learned from COVID-19. Overall, these findings highlight the need for further research into the development of strategies to address changes in care-seeking behavior during access-limiting scenarios.

## INTRODUCTION

The coronavirus disease 2019 (COVID-19) pandemic dramatically impacted and burdened our healthcare systems, changing individual perceptions of our system and the way medicine is practiced. By the end of 2022, there had been a reported total of 1,095,224 COVID-19 related-deaths, and COVID-19 remained a top 10 leading cause of death in the United States.^1^,^2^ This widespread impact on public health likely led to significant changes in patient emergency department (ED) care-seeking behavior. Early in the pandemic, reports from emergency medical services (EMS) and EDs showed a general decrease in EMS responses, patient transports, and ED patient volume.^3^,^4^

Fear of exposure to the virus may have contributed to patients avoiding the hospital, even for life-threatening conditions. For example, a study in Hong Kong found that patients experiencing acute myocardial infarctions (MI) during the pandemic had longer symptom-to-first medical contact time, and more patients presented outside of the revascularization window.^5^ Similar trends were observed for strokes, with more patients experiencing severe strokes while fewer patients were within the window to receive tissue plasminogen activator.^6^, ^7^ These trends suggest that fear of COVID-19 may have outweighed the perceived need for medical care, leading patients to delay seeking treatment.

Some low- or intermediate-acuity patients may have avoided seeking care altogether, perceiving their symptoms as low severity, fearing COVID-19, or adhering to stay-at-home orders. One survey examining fears of the risk of COVID-19 exposure in hospitals revealed that 16.9% of respondents prioritized avoidance of COVID-19 exposure in the ED over seeking appropriate care for symptoms consistent with MI, and 25.5% avoided care for symptoms of appendicitis.^8^ The fear of hospital exposure to the virus likely affected patients’ perception of symptom severity, leading them to postpone seeking care. Increases in risk tolerance and delays in referral to the ED may have led to increased acuity and corresponded to worse clinical outcomes when compared to pre-pandemic rates.

Across the US and globally, ED visits and hospital admissions decreased regardless of the severity of complaints.^4^,^9^–^11^ However, reported data on whether the decline in patient volume during the pandemic was also associated with a higher mortality rate have varied. Some studies reported no statistically significant increase in observed in-hospital mortality during the early pandemic,^12^ while others reported greater incidence of acute kidney injury and lactic acidemia and increased incidence of early in-hospital mortality.^10^ In high-acuity cases, whether due to local COVID-19 restrictions or fear, delays to seeking care posed the potential to impact patient outcomes. For patients experiencing acute MIs or strokes, the adage “time is tissue” best emphasizes the time-sensitive nature of receiving treatment.

Our goal in this study was to better characterize the relationship between changes in patient volume and overall acuity independent of COVID-19 cases by analyzing data from a Level I trauma center in Southern California. By controlling for COVID-19 cases, we contribute to the body of literature that delineates whether the observed decrease in patient volume correlated with an increase in mortality rates. We hope to provide insight into the impacts of delayed care-seeking behavior, government restrictions, and healthcare system adaptations during the pandemic. We hypothesized that during the COVID-19 pandemic there were changes in ED utilization patterns that impacted patient acuity, as measured by admission rate, mortality rate, ED volume, Emergency Severity Index (ESI), and female:male ratio even when controlling for COVID-19 cases.

**Population Health Research Capsule**

What do we already know about this issue?

*The COVID-19 pandemic put a significant strain on the healthcare system through increased patient mortality, and it posed various barriers to accessing healthcare*.

What was the research question?

*Did changes in emergency department (ED) utilization during the pandemic affect patient acuity when controlling for COVID-19 cases?*

What was the major finding of the study? *Admission rose from 26.0% (25.6–26.3) to 28.0% (27.6–28.5) (P <.001) and mortality rose 0.89% to 1.17% (P <.001)*.

How does this improve population health?

*This study demonstrates shifts in ED utilization patterns and patient acuity, which contributes to knowledge that may support responses in future health crises*.

These measures provide quantitative evidence of the impact that changes in patient care-seeking behavior had on influencing changes in ED patient acuity during the pandemic. Understanding the effects of patient behavioral shifts and healthcare system adaptations is crucial for preparing for future large-scale public health crises and optimizing ED resource allocation and mobilization based on lessons learned from COVID-19.

## METHODS

The study was performed at the University of California, Irvine Medical Center, a Level I trauma center in Southern California with over 58,000 annual ED visits and over 6,000 annual trauma visits; trauma represents about 10% of the ED census. The ED serves a primarily urban population and also has a Level II pediatric trauma center designation. It is strategically located within 20 miles of two other Level II adult trauma centers and one Level I pediatric trauma center. We examined 97,793 ED visits queried from an administrative database comprised of anonymized datapoints from the electronic health record. Incomplete records were excluded listwise from the analysis, and no imputation methods were used. We have no reason to believe that the extent of missing data differed between the study periods. Data extraction was performed by the department’s data analyst, and no adjudication was required as missingness led to exclusion rather than disagreement. We included all patients who presented to the ED from January 2019–December 2020. We included that time frame to account for potential secular trends by calendar month. COVID-19 cases began presenting to the ED in March 2020. While we considered excluding January and February from both years to achieve symmetry in study periods, we concluded that the ability to compare trends between 2019 and 2020 in the absence of COVID-19 outweighed the benefits of having perfectly symmetrical durations.

Primary outcome measures were hospital admission rate and all-causes mortality rate among non-COVID-19 patients who presented to the ED. Secondary outcome measures included the mean number of ED visits per month, mean ESI, and female:male ratio among non-COVID-19 patients referred to the ED. We compared primary and secondary outcome measures before the pandemic (January 1, 2019–February 28, 2020) and during the pandemic (March 1–December 31, 2020). We made comparisons among all patients first, and then by excluding COVID-19 patients. This study received approval from the institutional review board (IRB) of the University of California, Irvine.

### Statistical Analysis

We used Pearson chi-square tests to compare hospital admission rate, mortality rate, and female:male ratio between the study periods. We also used Mann-Whitney tests to compare the mean number of ED visits per month, and mean ESI between the study periods. For multivariate analysis, we used the logistic regression analysis method to compare the primary outcomes between the study periods, adjusting for sex, age, ESI, and primary diagnosis. To control for the confounder effect of age, we calculated the mean age for the patients before the pandemic and during the pandemic and input them into the logistic regression model. To control for the confounder effect of primary diagnoses, we classified them into 96 distinct groups and presented them into the logistic regression model.

We did not include age and primary diagnosis in the study outcomes because our primary goal was to isolate for the effects of the pandemic and not to test for statistical significance in the differences of these values. The statistical analysis was repeated after excluding COVID-19 patients. and the results are presented separately. Odds ratio (OR) for the study period, P-value and Nagelkerke R^2^ are reported for the logistic regression analysis. Continuous variables are presented as mean ± standard deviation. Proportions are reported as percentages and 95% confidence intervals. P-values < .05 were considered statistically significant. We used IBM SPSS Statistics v27.0 (IBM Corp., Armonk, NY) for data analysis.

## RESULTS

The study sample included 97,793 ED visits by 61,303 patients from January 1, 2019–December 31, 2020. Of the total ED visits, 59,937 occurred before the pandemic period and 37,856 occurred during the pandemic. Of the pandemic period visits, 2,269 (6%) were related to COVID-19 (Table 1).

**Figure 1:**
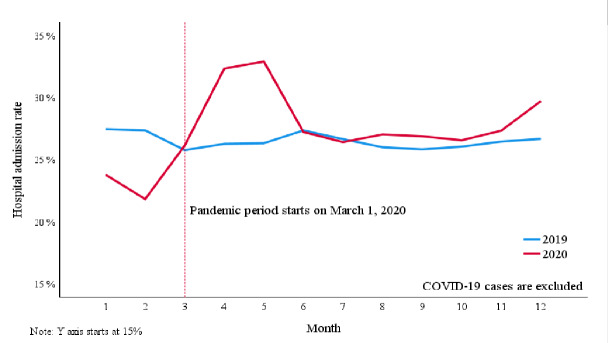
Hospital admission rates excluding COVID-19 cases following initial emergency department presentation, examining administrative data from January 1, 2019–December 31, 2020. This figure indicates the increase in hospital admission rate at the onset of the pandemic in March 2020 and the sustained elevation of admission rate compared to the year 2019, prior to the pandemic. The spike in admission rate is evidence of an increase in overall patient acuity throughout the time frame.

Hospital admission rate was 26.0% (25.6–26.3%) before the pandemic and rose to 32.3% (31.9–32.8%) during the pandemic (P < .001) (Table 1). After excluding COVID-19 cases, the hospital admission rate remained at 28.0% (27.6–28.5%), which was higher than before the pandemic (P < .001) (Figure 1). In logistic regression analysis, hospital admission was higher during the pandemic period compared to before the pandemic, adjusted for sex, age, ESI and primary diagnosis (OR 1.09, P < .001, Nagelkerke R^2^ 0.34). The association did not change by excluding COVID-19 cases (OR 1.09, P < .001, Nagelkerke R^2^ 0.39). The mean age of patients in the pre-pandemic period was 48.7 years (SD 19.13; median 48), and 48.4 years (SD 18.81; median 48) during the pandemic period.

**Figure 2:**
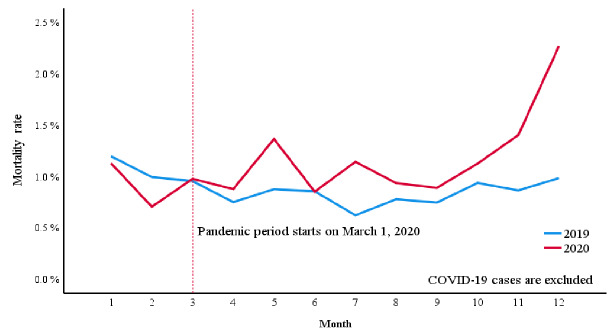
Overall emergency department mortality rate excluding COVID-19 cases before and during the COVID-19 pandemic, examining administrative data from January 1, 2019–December 31, 2020. The data show that at the onset of the pandemic in March 2020, mortality rate surged above the levels seen in 2019 and remained elevated throughout the remainder of the year.

During the study period, 239 patients died in the ED, 524 died after admission, and 249 died after discharge (Table 1). Before the pandemic, the overall mortality rate per ED visit was 0.8859% (.8125–.9642%). During the pandemic, the overall mortality rate per ED visit rose to 1.2706% (1.1602–1.3886%). After excluding COVID-19 cases, the overall mortality rate per ED visit remained elevated at 1.1746% (1.0652–1.2920%) when compared to the pre-pandemic period (Figure 2), representing a relative increase of 32.6% in mortality rate even after excluding COVID-19 cases. In logistic regression analysis, the pandemic was associated with higher mortality after adjusting for sex, age, ESI, and primary diagnosis (OR 1.293, P < .001, Nagelkerke R^2^ 0.35). The association did not change by excluding COVID-19 cases (OR 1.293, P < .001, Nagelkerke R^2^ 0.35).

**Figure 3:**
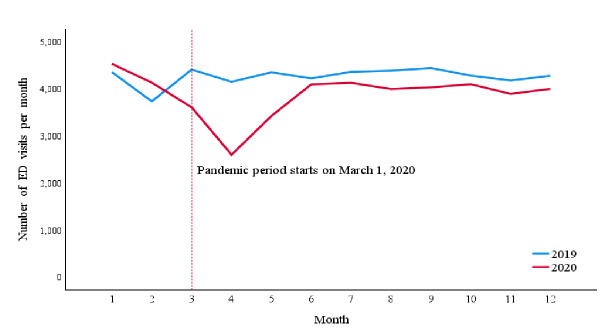
Number of emergency department visits per month excluding COVID-19 cases, examining administrative data from January 1, 2019–December 31, 2020. The data reveal a sharp decline in ED visits beginning in March 2020, coinciding with the onset of the pandemic. This decrease is sustained throughout the remainder of the year, with visits consistently lower in 2020 compared to 2019. *ED*, emergency department.

The mean number of ED visits per month was 4,271.2 ± 193.1 before the pandemic and decreased to 3,786 ± 476.8 per month during pandemic (P = .01). By excluding COVID-19 cases, the mean number of ED visits during the pandemic further decreased to 3,558.7 ± 437.1, which was significantly less than the pre-pandemic period (P < .001) (Figure 3). Trauma represented 12.62% (N = 7,567) of ED visits before the pandemic and represented 12.1% (N = 4,608) of ED visits during the pandemic. While not included in the statistical analysis, supplemental data on the breakdown of trauma-related presentations can be found in the appendix (Table S1).

**Figure 4:**
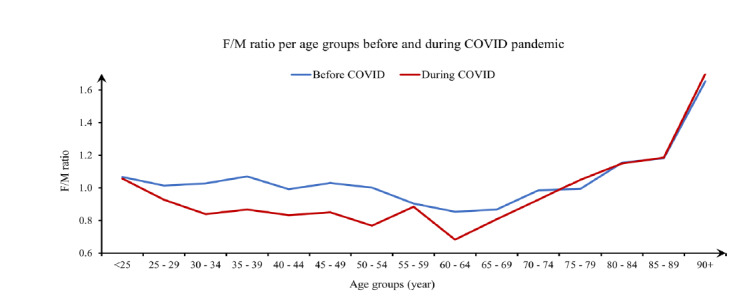
Female:male ratio for patients presenting to an emergency department before and during the COVID-19 pandemic, categorized by age groups, examining administrative data from January 1, 2019–December 3, 2020. The data reveal a consistent decrease in the female:male ratio throughout the measured pandemic period (March 1–December 31, 2020). *F:M*, female:male ratio.

The mean ESI of 59,205 pre-pandemic ED visits was 2.85 ± 0.707. During the pandemic, the mean ESI of 35,200 non-COVID-19 ED visits was 2.84 ± 0.689 (P = .03). Figure 4 shows female:male ratio per different age groups before and during the pandemic. Overall, female:male ratio was 1.003 (1.003 – 1.004) before the pandemic and 0.885 (0.880 – 0.889) during the pandemic, excluding COVID-19 cases (P < .001) (Table 1).

## DISCUSSION

Our results indicate that the decrease in the number of visits during the pandemic was not limited to non-emergency cases; some patients in need of emergency care likely delayed seeking treatment. As a result, patient acuity increased as represented by higher admission and mortality rates, and the increase in mortality rate persisted even after excluding COVID-19-related cases. While the mean ESI demonstrated a statistically significant increase in patient acuity as well, this change was small and not clinically useful. The ESI does not accurately represent the increase in acuity in this period, which would be consistent with previous literature that ESI may not accurately represent acuity.^13^ With the limitations in the accuracy of ESI, ED leadership could consider using multiple measures of acuity in combination with ESI, or use more objective measures of acuity altogether. More objective systems have been proposed that have shown promise, such as early warning systems that predict urgency based on vital signs and level of consciousness scales.^14^ However, those systems would need to be validated and could potentially under-triage complaints with normal vital signs and level of consciousness such as giant cell arteritis or spinal cord injuries. Better systems would need to be developed and validated before any alternative to ESI could be recommended as a replacement.

The decline in overall number of ED visits during the pandemic aligns with other studies that demonstrated a 20–58% reduction in visits.^4^,^9^,^11^ However, the significant increases seen in patient acuity throughout the literature emphasizes the need to allocate resources properly in future health crises. Patient acuity refers to the intensity of care that a patient requires, and higher acuity places much greater demands on staff to provide both direct and indirect patient care. While not measured in this study, we acknowledge that changes to ED staffing, local protocols, and institutional policies during the pandemic may have influenced the observed outcomes.

In this study, we found that patient volumes decreased, and the mean ESI did not reflect a clinically useful change in acuity, despite the increased mortality and admission rates. This identifies the additional challenge of properly allocating resources during health crises, as it can be difficult to predict dynamic changes in patient acuity. To optimize an ED’s workforce, ensure maximum patient safety, and minimize mortality, it may be beneficial to consider metrics beyond patient volume and ESI when allocating staffing and resources. Measures could include the mortality rate, admission rate, or weighted diagnosis-related groups per month, although further research would be needed to identify and validate the best alternative measures of patient acuity to be used when determining resource allocation.

The significant decline in female:male ratio during the pandemic could reflect a difference in the ED use pattern between the sexes. One study conducted during the pandemic found that men were less likely to avoid ED visits (adjusted OR 0.53) compared to women.^15^ However, other studies reported no statistically significant difference in avoidance behaviors between the sexes or that men were more likely to avoid ED visits.^16^,^17^ In the absence of a universal trend in data from other EDs supporting our measured decline in female:male ratio, it is unclear what drivers contributed to this finding. This observed sex difference could indicate anything from regional variations in self-triaging behaviors to distributions in underlying health conditions among sexes or local differences in sex-based behaviors such as caretaking responsibilities. Self-triage in the context of this discussion is a patient’s attempt to assess the priority of seeking care for their current medical complaint. Multiple factors influence self-triage decisions, including perceived risk and fear, which may have played a role in patients’ care-seeking behavior during the pandemic.

Understanding factors that influence patient decisions to seek emergency care are of foremost importance to managing future large-scale emergencies. Emergency situations can lead to damaged infrastructure, impact the economy, or overwhelm available resources. During the pandemic, transportation challenges and stay-at-home orders were significant barriers to seeking care.^18^,^19^ Fear of COVID-19 appeared to be a major factor influencing patient decision-making and self-triage behaviors during the pandemic. As discussed, one survey demonstrated that up to a quarter of patients may have prioritized avoidance of COVID-19 exposure over seeking lifesaving care in an emergency.^8^ In such cases, when patients delay seeking care based on perceived risk, an associated increase in acuity is expected.

The increased mortality rate seen in our study could reflect such self-triage behaviors, especially since this increased mortality rate was maintained even when excluding COVID-19 patients. There is a concern that severely ill patients may not have sought care in a timely manner, whether due to fear or barriers. If an increase in risk-taking behavior and severity of disease is associated with a significant decline in ED inflow, it could prove beneficial for healthcare forces to provide additional outreach services, such as telemedicine, to maintain patient safety and health during future access-limiting situations.

Online tools guiding patients’ self-assessments might help optimize ED care-seeking decisions when patients choose to self-triage. Throughout the pandemic we saw the introduction of many novel algorithmic self-triage support tools ranging in complexity from healthcare worker flowchart screening to artificial intelligence chatbots integrated into existing health portals.^20^,^21^ Some of these were used on a large scale such as the US Centers for Disease Control and Prevention symptom-checker tool, and others were designed to target smaller populations such as college campuses.^20^ Multiple studies examining online symptom-checker tools observed trends of peak usage and/or symptom recognition preceding patient surges in local hospitals.^22^–^24^ It is likely that these tools encouraged sick patients to seek care, and further study of their data could yield the ability to anticipate healthcare demand and provide additional information. The future development of these tools could help combat behaviors that contribute to hospital avoidance and delays in seeking care in access-limiting situations.

Throughout the pandemic, another common form of outreach was telehealth. While it can be difficult for EDs to incorporate telehealth given the nature of emergency situations, EDs could focus on forward triage as a means of telemedicine outreach. University of California Irvine Health incorporated a telemedicine system to provide 24/7 increased access to primary care clinicians during the pandemic. We acknowledge that this may have affected the results of this study as lower acuity patients may have sought care through alternative methods being offered within the same health system. Some studies have demonstrated feasibility and success with forward-triage.^25^–^28^ With an increase in barriers to care, stay-at-home mandates, and patient fears, telemedicine could help to increase access for lower acuity patients who are seeking care for non-life-threatening concerns. However, many challenges have been identified in the implementation of telemedicine, such as technical, legal, and ethical issues.^29^ Further research is required to characterize the feasibility and the effectiveness of these telemedicine systems to address challenges in access-limited scenarios.

This study demonstrates significant shifts in patient acuity and mortality during the pandemic, which could hold implications that extend to natural disasters or other public health crises. Our findings highlight the need to anticipate and address changes in patient behavior and barriers to seeking care during public emergencies when fear of the virus, hospital avoidance, or access barriers delay care and worsen outcomes. These findings also provide valuable data that contribute to our understanding of how the COVID-19 pandemic affected our healthcare system. Forms of outreach described here could be considered in future access-limiting situations to maintain patient safety. However, future research is needed to develop strategies that address the challenges posed by changes in care-seeking behaviors and local government restrictions to ensure minimal impact on patient mortality during future crises.

## LIMITATIONS

This study is subject to the known limitations of a before-after study design. The study design may be limited by temporal confounding in which other unseen factors that occurred during the study period could have influenced the outcomes of the study, making it difficult to attribute the outcomes solely to the COVID-19 pandemic. Before-after study designs are also limited to discrete periods in time, and if initial measurements were different, the differences observed may represent a regression to the mean. Additionally changes to the methods of measurement may also occur during the period that data was being recorded, which can influence results. We addressed patient diagnosis variability by categorizing primary diagnoses into 96 groups and adjusting for them in the multivariate analysis, but residual confounding may persist due to imperfect adjustment. Lastly, the single-center design of the study makes it subject to specific local policies, staffing changes, and pandemic response strategies, which limit generalizability.

## Supplementary Information



## Figures and Tables

**Table 1:** Study samples before and during COVID-19 at a Southern California hospital, including and excluding COVID-19 cases. The data from January 1, 2019–December 31, 2020 includes both the primary outcome measures of hospital admission rate and all-causes mortality rate, and the secondary outcome measures of female:male ratio and Emergency Severity Index.

Variable	Category	Study Period					

		Before Pandemic		During Pandemic			

				Including COVID-19		Excluding COVID-19	

		Count	%	Count	%	Count	%
Sex	Female	30,013	50.1%	17,790	47.0%	16,703	46.9%
	Male	29,921	49.9%	20,066	53.0%	18,884	53.1%
Hospital admission	No	44,365	74.0%	25,613	67.7%	25,613	72.0%
	Yes	15,572	26.0%	12,243	32.3%	9,974	28.0%
Emergency Severity Index	1	577	1.0%	402	1.1%	395	1.1%
	2	17,838	30.1%	10,595	28.3%	10,195	29.0%
	3	31,269	52.8%	21,014	56.1%	19,655	55.8%
	4	9,087	15.3%	5,110	13.6%	4,647	13.2%
	5	434	0.7%	333	0.9%	308	0.9%
Death in ED	No	59,809	99.8%	37,745	99.7%	35,479	99.7%
	Yes	128	0.2%	111	0.3%	108	0.3%
Death after admission	No	59,648	99.5%	37,621	99.4%	35,395	99.5%
	Yes	289	0.5%	235	0.6%	192	0.5%
Death after discharge	No	59,823	99.8%	37,721	99.6%	35,469	99.7%
	Yes	114	0.2%	135	0.4%	118	0.3%

*ED*, emergency department.
