# Supplementary material for: Emergency Department Utilization and Patient Acuity in the Setting of Care-Seeking Hesitancy: Insights from the COVID-19 Pandemic
Source: West J Emerg Med. 2025 Sep 25;26(5):1217–25. doi: 10.5811/westjem.43530 (PMC12591650; doi:10.5811/westjem.43530)

**APPENDIX**

**Table S1.** Demonstrates the number of trauma related emergency department visits before and during the COVID-19 pandemic at a hospital in Southern California, examining administrative data from 1/1/2019 to 12/31/2020. Trauma related visits are categorized by mechanism of injury.


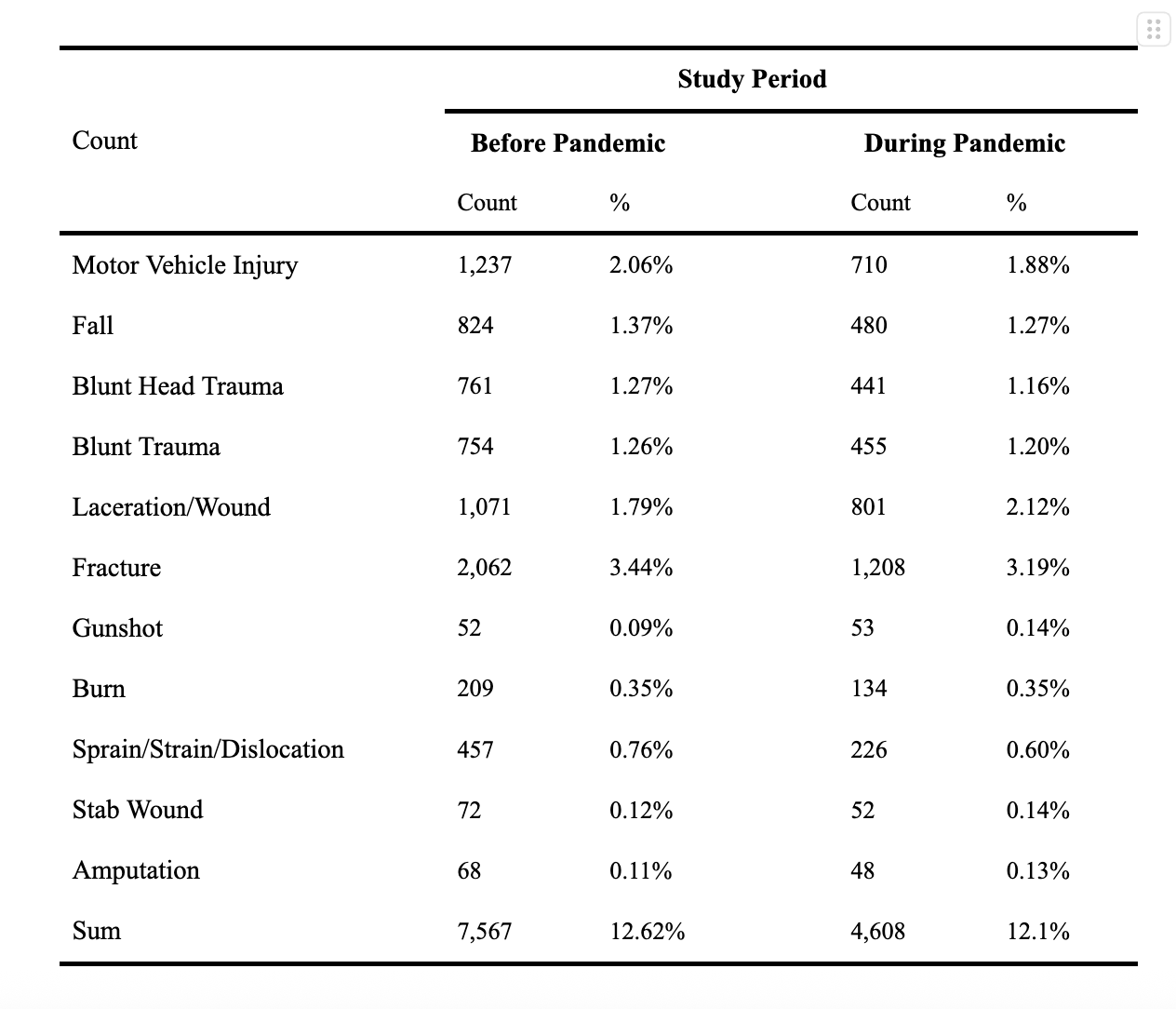

Supplement: Supplementary file 1 [file wjem-26-1217-s001.docx]
